# Carglumic Acid Treatment of a Patient with Recurrent Valproic Acid-induced Hyperammonemia: A Rare Case Report

**DOI:** 10.7759/cureus.3292

**Published:** 2018-09-12

**Authors:** Yasar Sattar, Saad Wasiq, Waqas Yasin, Ali M Khan, Mahwish Adnan, Shristi Shrestha, Nirav B Patel, Sharaad Latchana

**Affiliations:** 1 Internal Medicine, Icahn School of Medicine Mount Sinai, New York, USA; 2 Psychiatry, University of Health Sciences, Islamabad, PAK; 3 Psychiatry, Medical College of Wisconsin Affliated Hospitals, Milwaukee, USA; 4 Psychiatry Resident, University of Texas Rio Grande Valley, Harlingen, USA; 5 Center for Addiction and Mental Health, University of Toronto, Toronto, CAN; 6 Public Health, State University of New York, Brooklyn, USA; 7 Department of Medicine, Lasante Health, New York, USA; 8 Medical Student, American University of Integrative Sciences, Bridgewater, BRB

**Keywords:** valproic acid, carglumic acid, carbaglu, viha, hyper ammonia, hyperammonemia

## Abstract

Valproic acid, first manufactured as an anticonvulsant, is commonly used to treat both neurological and psychiatric conditions. A rare and deadly side effect of this medication is hyperammonemia, presenting as lethargy, confusion, seizure, and, ultimately, coma. In rare circumstances, hyperammonemia can be recurrent and devastating, especially in patients with an underlying N-acetyl glutamate synthase (NAGS) deficiency, as the valproic acid can enhance this enzyme deficiency and inhibit the conversion of ammonia into urea in the liver. For these subtypes of patients, the United States Food and Drug Administration (US FDA) has recently approved carglumic acid, a medication that can act as a scavenger by effectively increasing the levels of NAGS, ultimately enhancing the conversion of ammonia to urea. In our case report, we have mentioned a patient with treatment-resistant bipolar disorder, who presented with elevated ammonia levels secondary to valproic acid treatment. Valproic acid was the only drug that was effective in his case, so we initiated therapy to reduce his elevated ammonia levels. After a thorough evaluation, we found the patient had a genetic NAGS deficiency. Carglumic acid was initiated and proved efficacious in our patient.

## Introduction

Valproic acid (VA) was first manufactured in 1881 and has been used therapeutically since 1962 [1]. VA is commonly used in the treatment of certain neurological and psychiatric conditions like epilepsy, migraine, bipolar disorder, and borderline personality disorder, and VA is sometimes used to treat patients with alcohol withdrawal. VA is also very effective in the reduction of psychotic symptoms like agitation and aggression in patients with traumatic brain injuries [2-3]. VA is generally considered safe and is part of the World Health Organization’s (WHO's) list of essential medicines [4]. Despite its safety profile, VA’s common side effects include dry mouth, nausea, and vomiting. One of the rare, but potentially life-threatening side effect of VA is hyperammonemia [5]. VA-induced hyperammonemia usually presents as lethargy, vomiting, or any focal neurologic deficit [6] and is usually due to VA’s toxic metabolite-induced inhibition of N-acetyl glutamate synthetase (NAGS), a rate-limiting enzyme of the urea cycle. This inhibition leads to increased ammonia levels [7]. VA-induced hyperammonemia can be recurrent and devastating, especially in patients with a genetic NAGS deficiency (NAGSD). We present a case of a patient diagnosed with recurrent hyperammonemia due to VA and describe the successful treatment of that patient.

## Case presentation

A 23-year-old African American man with a history of bipolar disorder presented to the Comprehensive Psychiatric Emergency Program with an altered mental state and copious vomiting. As per the emergency protocols, two large-bore (16 gauge) intravenous cannulas were placed, and a standard saline infusion was started to treat the patient’s descending blood pressure. The initial examination revealed a Glasgow Coma Scale Score of 15 (eyes, 4; verbal, 5; and motor, 6), and a quick neurological exam failed to reveal any deficits in the extremities. The patient exhibited normal muscle strength, deep tendon reflexes, and cranial nerve function. His gait could not be assessed due to fatigue and the emergency condition. Table 1 presents the clinical laboratory values at admission.

**Table 1:** Clinical laboratory values on presentation Abbreviations: HCO_3_, bicarbonate; pCO_2_, partial pressure of carbon dioxide.

Analyte	Result
Hemoglobin	121 g/L
Platelet count	170 x10^9^/L
Peripheral blood smear	Elevated neutrophil count
C-reactive protein	15.23 nmol/L
pH	7.50
pCO_2_	3.72 kPa
HCO_3_	36 mmol/L
O_2_ Saturation	90%
Lactate	0.955 mmol/L
Stool culture results	Negative
Urine culture results	Negative
Blood culture results	Negative

The patient’s medication log stated he was currently taking VA 1500 mg daily, split into a 500-mg morning dose and a 1000-mg evening dose. His medical records revealed this VA dose was initiated four months prior after trials with other medications had failed due to adverse effects (diarrhea due to lithium). After starting VA, the patient was monitored via follow-up examinations in the clinic on a monthly basis. On his second-month follow-up visit, the patient reported concerns of weakness and fatigue. Laboratory tests revealed elevated ammonia and VA levels. At this time, the patient was diagnosed with VA-induced hyperammonemia. The VA treatment was stopped, and the patient was started on lactulose syrup and lamotrigine. We monitored the patient weekly. After his third weekly visit, he reported concerns of worsening of manic symptoms and severe bullae and rashes on his chest. The lamotrigine was stopped and manic symptoms recurred, leading to reinstating the VA treatment with weekly follow-up monitoring. His ammonia levels were elevated on all follow-up visits. A daily combination of lactulose syrup and levocarnitine was added to the treatment regimen in each weekly visit, but his ammonia levels continued to rise.

The patient presented with neurological symptoms with vomiting. Given his medical records, he was diagnosed with VA-induced hyperammonemic encephalopathy. His serum ammonia level was 68 µmol/L (reference range, 11 to 32 µmol/L). His serum VA level was within the reference range. After successful symptomatic control, the patient tested positive for an underlying genetic NAGSD. Therefore, we concluded this patient’s exaggerated response to VA in developing hyperammonemia was related to his underlying NAGSD. He was discharged from the inpatient facility with a daily dosing of 1500 mg VA and 800 mg carglumic acid.

## Discussion

VA is a short, branched-chain fatty acid that undergoes beta-oxidation in the mitochondria of hepatocytes and is converted into the toxic metabolite valproyl CoA. This metabolite depletes N-acetyl glutamate by the inhibition of N-acetyl glutamate synthetase, a rate-limiting enzyme [7]. VA can effectively treat a variety of neurologic and psychiatric conditions and is generally considered to be safe within a broad therapeutic range [8]. However, in patients with an underlying genetic deficiency of NAGS, VA can cause recurrent hyperammonemia, a fatal condition that can present as any focal neurologic deficit. An inherited NAGSD is an extremely rare urea cycle disorder with an estimated incidence of less than one in 2,000,000, caused by recessive mutations in the NAGS gene. Mitochondrial NAGS synthesizes the allosteric activator of the first enzyme of the urea cycle, carbamoyl phosphate synthetase 1 (CPS1). Clinically and biochemically, NAGSD is indistinguishable from a CPS1 deficiency, and common biochemical features include increased amounts of plasma ammonia and glutamine, reduced plasma citrulline, and normal or low levels of urinary orotic acid. NAGS enzyme assays are problematic, as they necessitate a liver specimen, so molecular analysis is the gold standard test for confirming NAGSD [9-10].

VA-induced hyperammonemia can present with normal or elevated drug levels [11]. We found multiple studies reporting the association of high ammonia levels with the use of VA. Twenty percent to 50% of patients who were prescribed VA had elevated ammonia levels [12]. A VA-induced rise in ammonia levels can be asymptomatic or clinically symptomatic. Physicians should rule out any underlying causes of elevated ammonia levels such as urea cycle disorders or drug-induced spikes. The diagnosis of VA-induced hyperammonemia can be made by a combination of clinical symptoms, elevated ammonia levels, normal or elevated VA levels, and urea cycle enzyme assay levels, as shown in Table 2.

**Table 2:** Summary of diagnosis criteria of VA-induced hyperammonemia Abbreviations: VA, valproic acid.

Symptoms	
	Abdominal Pain
	Vomiting
	Lethargy
	Confusion
	Stupor
	Seizure
	Focal neurological deficits
	Coma
Diagnosis	
Serum Ammonia Level	Elevated (reference range, 11–32 µmol/L)
Serum Valproic Acid Level	Can be in the reference range or elevated (reference range, 346–693 µmol/L)
Differentials	
	Rule out urea cycle disorders

**Figure 1:**
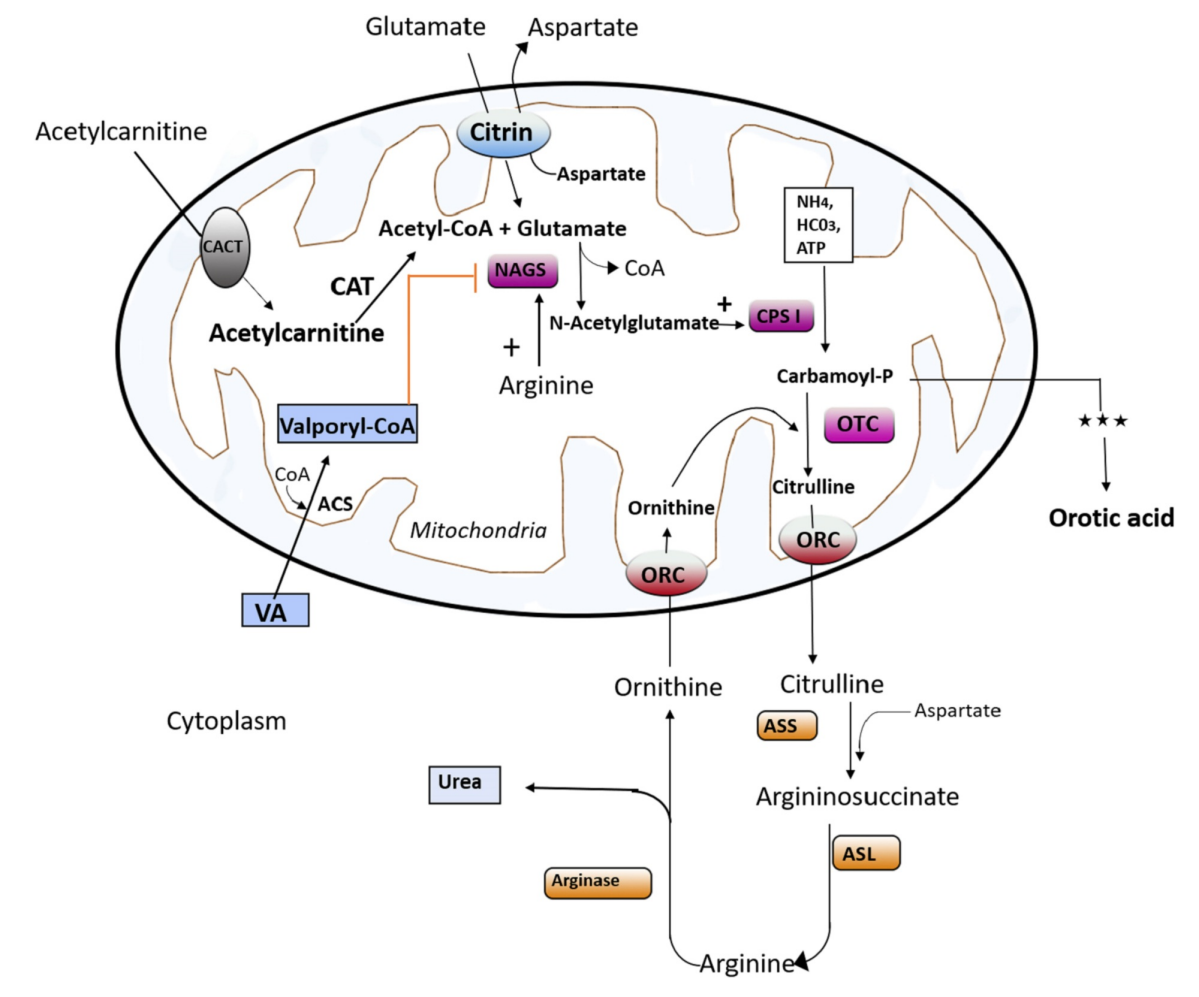
Summary of how NAGSD affects the urea cycle in VA-induced hyperammonemia Abbreviations: NAGSD, N-acetyl glutamate synthase deficiency; VA, valproic acid; CACT, carnitine-acylcarnitine translocase; CAT, chloramphenicol acetyltransferase; CoA, coenzyme A; NAGS, N-acetyl glutamate synthase; CPS, carbamoyl phosphate synthetase; ATP, adenosine triphosphate; ORC, ornithine carrier protein; ASL, argininosuccinic lyase; ASS, argininosuccinate synthase.

For both asymptomatic and symptomatic ammonia level elevations, consider a discontinuation of the medication and add alternative medication to control psychiatric symptoms [13]. VA-induced hyperammonemia and how NAGSD affects the whole urea cycle is summarized in Figure 1 [14].

**Figure 2:**
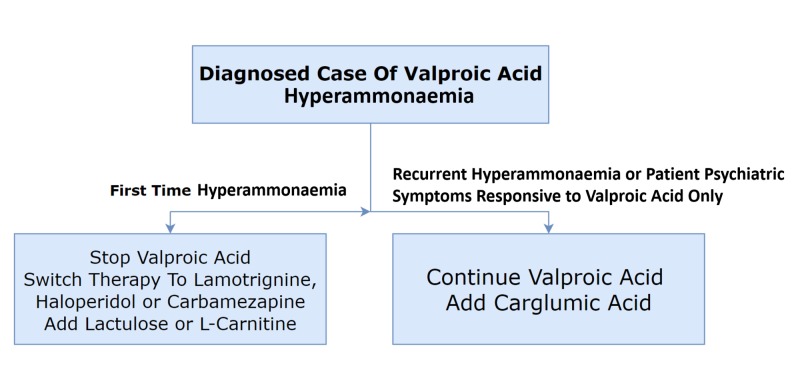
The management of a patient with VA-induced hyperammonemia Abbreviation: VA, valproic acid.

In patients with a congenital deficiency of NAGS, NAGS inhibition by VA is more pronounced and can cause recurrent hyperammonemia in patients taking VA. The US FDA approved carglumic acid for use in patients with an inherited deficiency in pediatric or adult patients. Carglumic acid is a great therapeutic option if the patient is only responsive to VA for psychiatric symptoms because the options for alternative medication are limited. Carglumic acid is generally safe and efficacious in these patients, but in some patients, it can cause adverse gastrointestinal effects like nausea, vomiting, and abdominal pain. Figure 2 summarizes the management of a patient with VA-induced hyperammonemia.

## Conclusions

Congenital urea cycle disorders should be ruled out in diagnosing VA-induced hyperammonemia. An early recognition of elevated ammonia levels can lead to a decrease in mortality or morbidity issues.

## References

[1] Scott DF (1993). The History of Epileptic Therapy: An Account of How Medication Was Developed.

[2] Showalter PEC, Kimmel DN (2000). Agitated symptom response to divalproex following acute brain injury. J Neuropsychiatry Clin Neurosci.

[3] Wroblewski BA, Joseph AB, Kupfer J, Kalliel K (1997). Effectiveness of valproic acid on destructive and aggressive behaviours in patients with acquired brain injury. Brain Inj.

[4] (2016). World Health Organisation. WHO Model List of Essential Medicines, 19th List. 2015.

[5] Tang JY, Kiang TKL, Ensom MHH (2017). Pharmacokinetic interactions between valproic acid and lorazepam (PIVOtAL study): a review of site-specific practices. Can J Hosp Pharm.

[6] Sousa C (2013). Valproic acid-induced hyperammonemic encephalopathy - a potentially fatal adverse drug reaction. SpringerPlus.

[7] Aires CC, van Cruchten A, Ijlst L, de Almeida IT, Duran M, Wanders RJ, Silva MF (2011). New insights on the mechanisms of valproate-induced hyperammonemia: inhibition of hepatic N-acetylglutamate synthase activity by valproyl-CoA. J Hepatol.

[8] Dixit S, Namdeo M, Azad S (2015). Valproate induced delirium due to hyperammonemia in a case of acute mania: a diagnostic dilemma. J Clin Diagn Res.

[9] Summar ML, Koelker S, Freedenberg D, Le Mons C, Haberle J, Lee H-S, Kirmse B (2013). The incidence of urea cycle disorders. Mol Genet Metab.

[10] Morizono H, Caldovic L, Shi D, Tuchman M (2004). Mammalian N-acetylglutamate synthase. Mol Genet Metab.

[11] Mojumder DK, De Oleo RR (2014). Differential ammonia decay kinetics indicates more than one concurrent etiological mechanism for symptomatic hyperammonemia caused by valproate overdose. Indian J Pharmacol.

[12] Dealberto MJ (2007). Valproate-induced hyperammonaemic encephalopathy: review of 14 cases in the psychiatric setting. Int Clin Psychopharmacol.

[13] Twilla JD, Pierce AS (2014). Hyperammonemic encephalopathy due to valproic acid and topiramate interaction. Case Rep Psychiatry.

[14] Aiyer R, Seide M, Stern RG (2016). Valproic acid induced hyperammonemia in a long time treated patient. Case Rep Psychiatry.

